# Hospital-acquired *Clostridium difficile* infection in Mainland China: A seven-year (2009–2016) retrospective study in a large university hospital

**DOI:** 10.1038/s41598-017-09961-0

**Published:** 2017-08-29

**Authors:** Qiaomai Xu, Yunbo Chen, Silan Gu, Tao Lv, Beiwen Zheng, Ping Shen, Jiazheng Quan, Yunhui Fang, Lanjuan Li

**Affiliations:** State Key Laboratory for Diagnosis and Treatment of Infectious Diseases, Collaborative Innovation Center for Diagnosis and Treatment of Infectious Diseases, The First Affiliated Hospital, College of Medicine, Zhejiang University, Hangzhou, 310003 Zhejiang China

## Abstract

*Clostridium difficile* infection (CDI) is associated with risk for severe disease and high mortality. Little is known about the extent of hospital-acquired CDI in Mainland China. In this study, we aimed to investigate the annual CDI incidence, bacterial genotypes, risk factors for severe CDI and survival over a 7-year period. A total of 307 hospital-acquired CDI patients were enrolled, and 70.7% of these cases were male. CDI incidence was 3.4 per 10,000 admissions. Thirty-three different sequence types (STs) were identified, among which ST-54 (18.2%), ST-35 (16.6%) and ST-37 (12.1%) were the most prevalent. During the follow-up period, 66 (21.5%) patients developed severe CDI and 32 (10.4%) patients died in 30 days. Multivariate analysis revealed that bloodstream infection, pulmonary infection and C-reactive protein were significantly associated with severe CDI. After adjustment for potential confounders, old age, bloodstream infection, fever, mechanical ventilation, connective tissue disease, macrolide use and hypoalbuminaemia were independently associated with 30-day mortality in patients with CDI. The CDI prevalence has been low and stable in our center, and STs of *Clostridium difficile* were different from dominant STs in Western countries. Our data emphasize the need of continued education and surveillance of CDI to reduce the CDI burden in China.

## Introduction

*Clostridium difficile* infection (CDI) is increasingly common in health-care facilities, and represents 20–30% of antibiotic-associated diarrhea^1^. Clinical CDI manifestations vary from mild diarrhea to severe complications of pseudomembranous colitis, toxic megacolon and death. Outbreaks in North America and Europe coincided with the emergence of a hypervirulent strain, NAP1/BI/027^2^. This fluoroquinolone-resistant strain produces not only the two usual toxins (toxin A and B), but also a third toxin (binary toxin, CDT); and this was frequently associated with high risk for poor prognosis. However, additional strains were also associated with increased CDI incidence and unfavorable outcomes, and CDI is becoming a persistent challenge throughout the world. Overall, CDI incidence has exceeded that of methicillin-resistant Staphylococcus aureus (MRSA), and amounts to 12.1% of all health care-associated infections in America^3, 4^.

To meet the CDI challenge, several countries have sought to monitor CDI nationally; and many studies have analyzed CDI isolates to determine the molecular epidemiology of *Clostridium difficile* (*C. difficile*) in different regions of the world^5–8^. However, awareness and surveillance of CDI in Asia have remained poor^9, 10^. Limited studies performed in Asia over the recent decade indicate that CDI is a significant cause of nosocomial diarrhea in this region, but the true prevalence of CDI remains unknown. Few studies have also reported CDI in China, and the majority of these studies were focused on antimicrobial resistance or sporadic case reports^11, 12^. Due to the lack of awareness of increased CDI frequency, long-term clinical characteristics, prognosis and risk factors, clinicians may overlook or misdiagnose CDI in Mainland China. Considering the enormous differences in geographical location, genetics of a population, diet habits and even antibiotic use, we expected a different picture of CDI in Mainland China, compared with reports from Western countries.

To understand CDI in our center, we initiated this study and retrospectively analyzed the annual CDI incidence, bacterial genotypes and risk factors for severe CDI over a 7-year period.

## Methods

### Patients and Definitions

This retrospective study was carried out at The First Affiliated Hospital, Zhejiang University School of Medicine, which is a 2,500-bed tertiary teaching hospital in Hangzhou, Zhejiang, China. From September 1, 2009 to September 30, 2016, inpatients with confirmed hospital-acquired CDI were enrolled and followed up for at least 30 days. Patients aged eighteen years or older were included in the study. Stool samples were collected after written informed consent was obtained from all of the patients. This study was performed in accordance with the declaration of Helsinki and approved clinical practice guidelines and was approved by the Medical Ethics Committee of the First Affiliated Hospital, Zhejiang University School of Medicine.

Diarrhea was defined as three or more loose stools within 24 hours. Inpatients with diarrhea, whose stool samples were positive for both *C. difficile* culture and toxin gene tests, were diagnosed as CDI. Hospital-acquired CDI is defined as CDI diagnosis established 48 hours after admission, or within 28 days after discharge^13, 14^. A CDI case was classified as severe if at least one of the following specific manifestations is fulfilled: severe colitis or a complicated course of disease, with significant systemic toxin effects and shock (severe or bloody diarrhea, severe abdominal pain, vomiting, ileus, temperature >38.9 °C, white-cell count >20,000/mm^3^, albumin level <2.5 mg/dL, and acute kidney injury); resulting in admission to the Intensive Care Unit (ICU), colectomy, or death within 14 days^15^. Patients with CDI recurrences (defined as new episodes within eight weeks after the confirmation of the first positive sample) were also excluded^16^.

### Microbiological data

Stool samples (semi-formed, unformed, or liquid) were submitted to the clinical microbiology laboratory and cultured anaerobically on cycloserine–cefoxitin–taurocholate agar (CCFA-TA; Oxoid) supplemented with 7% sheep serum at 35 °C for 48 hours. Strains were confirmed by matrix-assisted laser desorption ionization-time of flight (MALDI-TOF) mass spectrometry using the Microflex LT system (Bruker Daltonik). The *tcdA*, *tcdB*, *cdtA* and *cdtB* genes were detected by PCR in all strains, as previously described^17, 18^. Multilocus sequence typing (MLST) with seven housekeeping genes (*adk, atpA, dxr, glyA, recA, sodA* and *tpi*) was performed on all toxigenic isolates, as previously described^19^. DNA sequences were submitted to the MLST database (http://pubmlst.org/cdifficile/) to assign the sequence types (STs).

### Data Collection

The following data of all patients with hospital-acquired CDI were collected through the hospital database, including age, gender, date of onset of diarrhea, prior underlying diseases, prior medication, prior surgery, clinical data, laboratory parameters, in-hospital medications and prognosis. “Prior” refers to within two months before the CDI diagnosis. CDI characteristics at enrollment were also collected. Vital signs and laboratory findings within 12 hours before or 24 hours post-enrollment were tabularized. Charlson comorbidity index score was used to evaluate the basic conditions of underlying diseases^20^. Follow-up clinical data were initiated upon hospital admission. For discharged patients, prognostic information was obtained from medical records, telephone contact, or personal visits. The primary endpoint of the study was 30-day mortality.

### Statistical Analysis

Annual CDI incidence was calculated as the number of events per 10,000 admissions. For the assessment of risk factors for severe CDI and 30-day mortality, continuous variables were expressed as median with interquartile range (IQR) or mean ± standard deviation (SD). Continuous data were compared in the univariate analyses by Student *t*-test or Mann-Whitney’s *U*-test. Nominal variables were expressed as number/percentage and compared using chi-square test. A Cox’s proportional hazard model was used to identify the predictors of time-dependent death of CDI patients. Candidate variables (*P* < 0.10) after a bivariate analysis were entered into a multivariate Cox’s model using a backward-forward approach. The survival curves of the different ST groups were plotted through the multivariate Cox’s model. In addition, a multivariate logistic regression analysis was performed to assess risk factors for severe CDI, in which the entry and removal probability for the stepwise-backward method was set as 0.05 and 0.10, respectively, and variables with *P* < 0.05 were retained in the final model. Hosmer-Lemeshow goodness of fit test was performed for logistic regression. Statistical analyses were performed using SPSS (version 23.0; SPSS Inc., Chicago, IL, USA).

## Results

### Incidence

**Figure 1:**
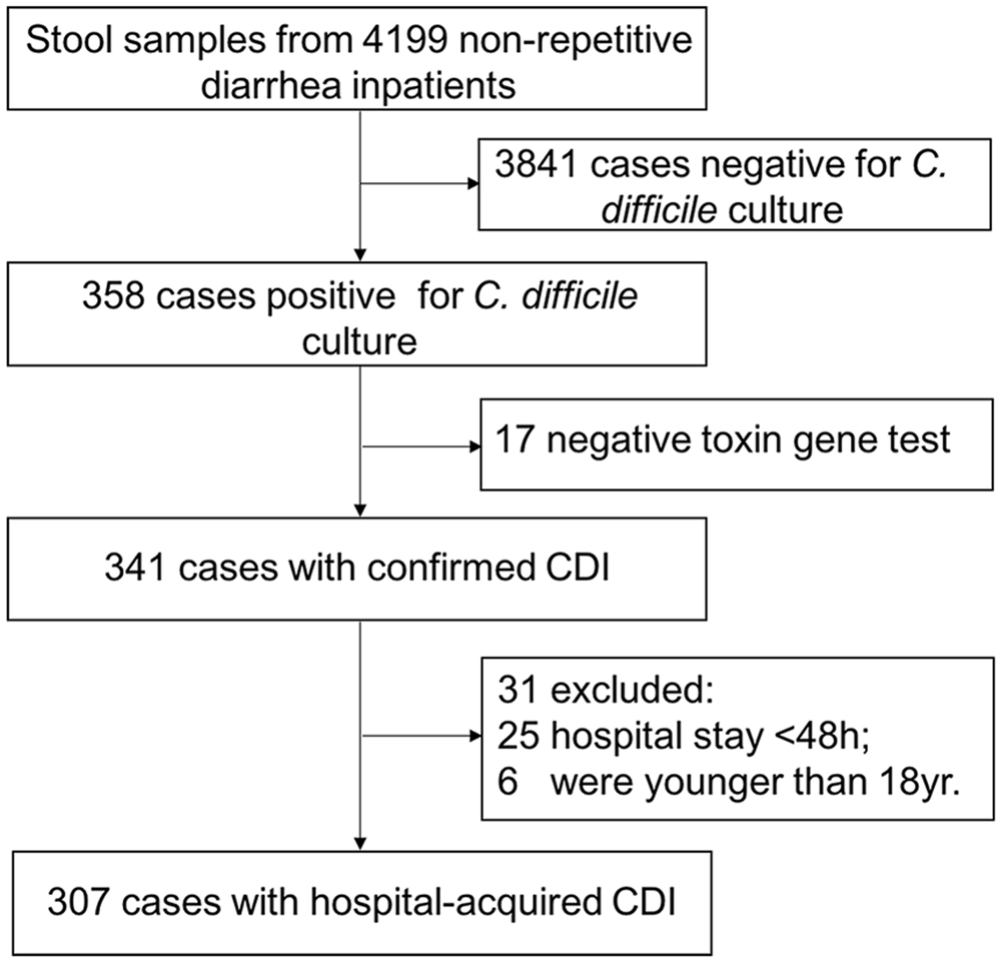
Flow diagram of the procedures used to identify hospital-acquired CDI patients.

During the 85-month study period, 307 of 0.91 million hospital admissions were diagnosed with hospital-acquired CDI (Fig. 1). The average incidence was 3.4 per 10,000 admissions (3.1 per 100,000 patient-days), which varied between 2.5 and 4.3 per 10,000 admissions annually. The average positive rate of toxigenic *C. difficile* in diarrhea patients was 7.3%, which varied between 5.2% and 8.9% annually (Supplementary Table 1).

### Patient Characteristics

The age of patients in this cohort ranged between 18 and 97 years (median age: 56 years). Male accounted for 70.7%. Median hospital stay was 30 days (interquartile range [IQR], 18–61 days). Both median duration of hospitalization before and after CDI onset was 14 days (IQR, 6–29; 7–31 days). The most frequent comorbidities were liver disease (25.6%) and solid tumor (24.1%). Within eight weeks prior to enrollment, 219 patients (70.0%) were admitted to the hospital at least once, and 16.0% of them had a history of ICU admission prior to developing CDI. The 203 patients (66.1%) received at least 1acid suppression medication, while antibiotic use was noted in 257 patients (83.7%), which were most commonly cephalosporins (50.2%), fluoroquinolones (32.6%), or β-lactam/β-lactamase inhibitors (29.6%). Furthermore, only 110 patients (35.8%) were treated with metronidazole (intravenous or oral) or oral vancomycin that targeted CDI. As the lack of understanding of CDI among clinicians, nearly half of these patients (*n* = 142, 46.3%) only received antidiarrheal and probiotics treatment; and the remaining 55 patients (17.9%) were not treated at all (Table 1).

**Table 1:** Demographic and clinical characteristics of patients with hospital-acquired *Clostridium difficile* infection.

Patient characteristics	Value or No. (%, *N* = 307)
Age	56.77 ± 20.21
**Gender**	
Male	217 (70.7)
Female	90 (29.3)
**Area**	
Rural	152 (49.5)
Urban	155 (50.5)
Smoke	86 (28.0)
Alcohol intake	69 (22.5)
**Medication exposures prior to developing CDI** ^**a**^	
Proton pump inhibitor	203 (66.1)
Chemotherapy	58 (18.9)
Use of any antibiotic not directed at CDI	257 (83.7)
Fluoroquinolones	100 (32.6)
β-lactam/β-lactamase inhibitor combinations	91 (29.6)
Cephalosporin	154 (50.2)
Carbapenem	79 (25.7)
Aminoglycoside	10 (3.3)
Glycopeptides	41 (13.4)
Macrolide	5 (1.6)
Co-trimoxazole	24 (7.8)
Other antibiotics	23 (7.5)
Antifungal agent	61(19.5)
Charlson comorbidity index score, median (IQR)	2 (1–3)
Myocardial infarction	1 (0.3)
Congestive heart failure	6 (2.0)
Chronic obstructive pulmonary disease	18 (5.8)
Cerebrovascular disease	23 (7.5)
Peptic ulcer disease	5 (1.6)
Connective tissue disease	9 (2.9)
Diabetes	51 (16.3)
Chronic kidney disease	40 (13.0)
Dementia	5 (1.6)
Leukemia	52 (16.9)
Malignant lymphoma	10 (3.3)
Solid tumor	74 (24.1)
Liver disease	79 (25.7)
AIDS	6 (1.0)
Prior hospitalization	215 (70.0)
Length of hospital stay, days, median (IQR)	30 (18–61)
Length of hospital stay before CDI, days, median (IQR)	14 (6–29)
Length of hospital stay after CDI, days, median (IQR)	14 (7–31)
Surgery prior to developing CDI	63 (20.5)
Abdominal surgery prior to developing CDI	41 (13.4)
Mechanical ventilation prior to developing CDI	43 (14.0)
Intensive care unit admission prior to developing CDI	49 (16.0)
**Ward of admission at onset of CDI**	
Medical unit	43 (14.0)
Surgical unit	144 (46.9)
Intensive care unit	58 (18.9)
Haemato-oncological unit	22 (2.2)
Geriatric unit	40 (16.0)
Any infection concomitant to CDI	116 (37.8)
Bloodstream infection concomitant to CDI	21 (6.8)
Pulmonary infection concomitant to CDI	86 (27.7)
Other infection concomitant to CDI^b^	23 (7.5)
Fever >38·5 °C	90 (29.3)
Leukocyte (cells × 10^9^/L), median (IQR)	7.1 (4.0–10.7)
Haemoglobin (g/dL), median (IQR)	99 (79–116)
Platelet (10^9^/L), median (IQR)	158 (76–243)
Albumin (g/dL), median (IQR)	33.1 (29.0–37.9)
Creatinine (μmol/L), median (IQR)	58 (44–79)
Alanine transferase (u/L), median (IQR)	22 (13–45)
C-reactive protein (mg/L), median (IQR)	28.7 (10.7–68.1)
**Therapeutic management**	
No therapy	56 (17.9)
Symptomatic treatment	142 (46.3)
Vancomycin (oral)	42 (13.4)
Metronidazole (intravenous or oral)	54 (17.6)
Vancomycin (oral) and metronidazole (intravenous or oral)	15 (4.9)
**Outcome of CDI**	
Intensive care unit admission	12 (3.9)
Recurrence within 54 days	13 (4.2)
Development of severe CDI	66 (21.5)
30-day all-cause mortality	32 (10.4)

Interquartile range (IQR); *Clostridium difficile* infection(CDI); ^a^Prior was defined as within two months before the diagnosis of CDI; ^b^Abdominal infection = 12; urinary tract = 4; upper respiratory tract = 2; surgical site = 3; intracranial infection = 2.

### Microbiological Characteristics

A total of 307 toxigenic isolates, isolated from 4,199 stool samples, were available for detailed microbiological characterization. Seventy-two isolates (23.5%) were A− B+ CDT− strains, 13 (4.2%) isolates were A+ B+ CDT+ strains and the rest (222, 72.3%) were A+ B+ CDT− strains. Toxigenic *C. difficile* strains were analyzed by MLST and divided into 33 different STs. The most common STs are listed in Table 2. ST-37 (PCR-ribotypes, RT 017) and ST-81 accounted for 63.9% (46/72) and 18.1% (13/72) of the A− B+ strains, respectively. For CDT+ strains, nine of these isolates belonged to ST-5 (RT 023), two belonged to ST-201, and one belonged to ST-1 (RT 027) and ST-11 (RT 078). ST-54 (56/222, 25.2%, RT 012) and ST-35 (51/222, 23.0%, RT 046) represented the most prevalent A+ B+ isolate in this cohort. We also found a total of 17 nontoxigenic isolates, eight of these isolates belonged to ST-37, five belonged to ST-39 (RT 085), two belonged to ST-2 (RT 014), and one belonged to ST-35 and ST-26 (RT 140).

**Table 2:** 30-day mortality and disease severity stratified by age and sequence types.

Stratification	Total	Mortality	Severity
	% (*N* = 307)	% (*N* = 32)	% (*N* = 66)
**Age group, y**			
18–29	38 (12.4)	2 (6.3)	10 (16.7)
30–39	32 (10.4)	1 (3.1)	1 (1.5)
40–49	41 (13.4)	3 (9.4)	5 (7.6)
50–59	50 (16.3)	4 (12.5)	8 (12.1)
60–69	47 (15.3)	4 (12.5)	13 (19.7)
70–79	46 (15.0)	5 (15.6)	12 (18.2)
80–89	47 (15.3)	11 (34.4)	15 (22.7)
≥90	6 (2.0)	2 (6.3)	1 (1.5)
**Microbiological characteristics**			
54	56 (18.2)	8 (25.0)	10 (15.2)
35	51 (16.6)	6 (18.8)	9 (13.6)
37	46 (15.0)	4 (12.5)	10 (15.2)
3	37 (12.1)	1 (3.1)	6 (9.1)
2	19 (6.2)	2 (6.3)	5 (7.6)
81	13 (4.2)	2 (6.3)	5 (7.6)
8	10 (3.3)	1 (3.0)	3 (4.5)
5	9 (2.9)	2 (6.3)	3 (4.5)
39	9 (2.9)	2 (6.3)	1 (1.5)
102	9 (2.9)	0 (0)	2 (3.0)
Presence of either or both binary toxin genes in toxigenic isolates	13 (4.2)	2 (6.3)	4 (6.1)
Toxin A negative and toxin B positive strains in toxigenic isolates	72 (23.5)	8 (25.0)	17 (25.8)

Mortality and CDI severity were analyzed by age groups, respectively. Additionally, stratification displayed the 10 most frequently found sequence types (STs) among toxigenic isolates.

### Risk Factors for Severe CDI

Sixty-six of 307 CDI episodes were classified as severe CDI (21.5%). The multivariate analyses of risk factors for severe CDI are shown in Table 3 (Candidate variables were selected by univariate logistic regression, Supplementary Table 2). The P value of Hosmer-Lemeshow test is 0.792. In the multivariate logistic regression, concomitant bloodstream infection (OR: 8.48, 95% CI: 1.65–43.49; *P* = 0.010), pulmonary infection (OR: 6.03, 95% CI: 1.16–31.36; *P* = 0.033) and CRP ≥100 mg/L (OR: 2.93, 95% CI: 1.34–6.42; *P* = 0.007) remained significantly associated with severe CDI.

**Table 3:** Risk factors associated with severe hospital-acquired *Clostridium difficile* infection analyzed by multivariate logistic regression.

Variable	Univariate analysis		Multivariate analysis	
	OR (95% CI)	*P* value	OR (95% CI)	*P* value
Age ≥65	2.035(1.173–3.529)	0.011		
Ward of admission at onset of CDI		0.047		
Medical	Reference			
Surgical	0.904 (0.311–2.632)			
Intensive care	1.000 (0.287–3.488)			
Haemato-oncological	2.571 (0.818–8.080)			
Geriatric	0.783 (0.233–2.632)			
Fever >38·5 °C	2.313 (1.313–4.074)	0.004		
ICU admission prior to CDI	2.540 (1.313–4.915)	0.006		
Mechanical ventilation prior to CDI	2.869 (1.445–5.694)	0.003		
Infection concomitant to CDI	2.230 (1.284–3.875)	0.004		
Bloodstream infection concomitant to CDI	3.734 (1.511–9.228)	0.004	8.481 (1.654–43.491)	0.010
Pulmonary infection concomitant to CDI	2.379 (1.343–4.212)	0.003	6.026 (1.158–31.363)	0.033
Chronic kidney disease	2.226 (1.087–4.559)	0.029		
Antibiotic	2.215 (0.901–5.445)	0.083		
Cephalosporin	1.796 (1.035–3.118)	0.037		
Leukocyte ≥15 × 10^9^/L	3.125 (1.397–6.991)	0.006		
Serum creatinine increase >50%	2.218 (1.059–4.642)	0.035		
CRP ≥100 mg/L	3.352 (1.633–6.882)	0.001	2.931 (1.339–6.417)	0.007

OR, Odds ratio; CI, confidence interval; variables entering the multivariate logistic analysis were selected by univariate logistic regression (Supplementary Table 2).

### Risk factors for 30-Day Mortality (all causes)

**Figure 2:**
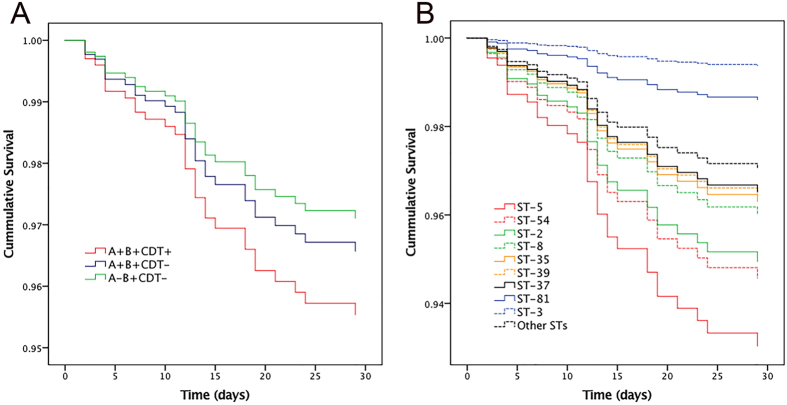
Mortality rates of all patients with *Clostridium difficile* infection up to 30 days after onset of diarrhea stratified by different toxins (**A**) or sequence types (**B**).

A total of 32 patients died within 30 days, accounting for 10.4% of the CDI population. The 30-day mortality increased with age, and the highest case fatality was observed among patients aged between 80 and 89 years (11/47, 23.4%) and >90 years (2/6, 33.3%). ST-5 (2/9, 22.2%) and ST-39 (2/9, 22.2%) were associated with higher 30-day mortalities (Table 2). A multivariate Cox’s model was performed to identify risk factors for 30-day mortality (Candidate variables were selected by bivariate analysis, Supplementary Table 3). Age ≥65 years (hazard ratio [HR]: 2.98, 95% CI: 1.27–7.02; *P* = 0.012), concomitant bloodstream infection (HR: 3.53, 95% CI: 1.50–8.32; *P* = 0.004), fever ≥38.5 °C (HR: 3.03, 95% CI: 1.45–6.31; *P* = 0.003), mechanical ventilation prior to CDI (HR: 3.20, 95% CI: 1.46–7.00; *P* = 0.004), connective tissue disease (HR: 5.53, 95% CI: 1.39–22.00; *P* = 0.015), macrolide use (HR: 10.17, 95% CI: 2.25–45.93; *P* = 0.003) and albumin ≤2.5 mg/dL (HR: 3.94, 95% CI: 1.38–11.25; *P* = 0.011) were independently associated with poor prognosis in CDI patients (Table 4). After adjustment of baseline differences by the multivariate Cox’s model, the 30-day mortality was found to be associated with changes in toxin profiles (Fig. 2A) and STs (Fig. 2B).

**Table 4:** Risk Factors associated with 30-day mortality analyzed by multivariable Cox’s proportional hazard mode in hospital-acquired *Clostridium difficile* infection patients.

Variable	B	Standard error	Wald chi-square	Hazard ratio	95% CI	*P* value
Age ≥65	1.092	0.437	6.251	2.981	1.266–7.017	0.012
Bloodstream infection concomitant to CDI	1.262	0.437	8.332	3.532	1.499–8.321	0.004
Fever >38·5 °C	1.108	0.375	8.725	3.027	1.452–6.312	0.003
Mechanical ventilation prior to developing CDI	1.164	0.399	8.491	3.201	1.464–7.002	0.004
Connective tissue disease	1.710	0.704	5.895	5.531	1.391–22.000	0.015
Macrolide	2.320	0.769	9.099	10.174	2.254–45.929	0.003
Albumin ≤2.5 g/dL	1.370	0.536	6.534	3.935	1.376–11.250	0.011

Statistical analysis was performed using a multivariable Cox’s proportional hazard model. Variables entering the multivariate analysis were selected by binary analysis (Supplementary Table 3).

## Discussion

In this retrospective study, a standard diagnosis procedure was used to determine hospital-acquired CDI frequencies between 2009 and 2016 in our center. Our analysis revealed an annually stable, low CDI prevalence in our center, and the average CDI rate was 3.4 per 10,000 admissions or 7.3% among diarrhea patients. We also analyzed the STs of all isolates, and the dominant STs were different from that reported in North America and Europe.

A recent meta-analysis of CDI incidence in Mainland China suggested that the pooled incidence of toxigenic *C. difficile* among patients with diarrhea was 14%, but it varied significantly from 23% to 3% in different regions^11^. A 8.4% (19/227) −15.6% (125/801) high CDI among diarrhea patients was reported in Western countries^21–27^. An average of 1.33 nosocomial CDI per 1,000 admissions (range: 0.74–2.30) was reported in 13 European hospitals^7^. The 7.3% CDI incidence among diarrhea patients or 3.4 per 10,000 admissions in this study was low.

We have reason to believe that a CDI diagnosis may have been missed in a portion of patients with diarrhea because of the poor awareness of CDI among physicians, representing the main reason for the relatively low CDI incidence. In addition, a portion of patients who developed CDI after discharge within 28 days were not included due to incomplete clinical data. Another possible reason was the different dominant CD strains compared in other countries.

Age appears to be important for developing CDI. The median age among patients in this study was 56 years, which was slightly younger than that reported in Western countries^6–8^. However, this study clearly revealed that the age composition of CDI patients was biased toward an older population: more than half of the cases were 50 years or older, and one in four cases was 70 years or older. The association of old age with CDI may suggest that weakened immunity is a condition for CDI.

Another finding in this study was that 70.7% of CDI patients were male, and 9.2% (20/217) of these male patients died within 30 days, which was in contrast with the findings reported in Western countries, where females were dominant among CDI patients^6, 7^. The population of CDI patients was also not predominant female in several studies from East Asian countries, we suggest that the difference in CDI patient gender may be related to geographical location, genetic disposition and diet habits^28–30^.

Furthermore, as high as 83.7% of CDI patients had a history of previous use of antibiotics in this study, which was in line with the rates reported by others^7, 27^, suggesting that the use of antibiotics may have caused imbalance of bacterial flora in the gut. It was interesting to note that the highest CDI occurred in patients admitted to the Internal Medicine Department, since these patients were most likely exposed to antibiotics for long periods and had a long hospital stay.

Due to the lack of awareness of CDI among physicians, the treatment of diarrhea did not follow the CDI treatment guidelines. Interestingly, when 30-day mortality was correlated with the treatment, mortality was 9.9% (14/142) in patients who accepted symptomatic treatment and 9.1% (5/55) in patients with no therapy; and both were lower than the overall mortality. The lower mortality in patients without treatment may suggest mild CDI, and only severe diarrhea brought the attention of physicians to possible CDI and initiated the CDI treatment. Furthermore, among patients who accepted symptomatic treatment, 100 of 142 (70.4%) patients received probiotics. Most of these probiotics contained *clostridium butyricum*, which has been shown to be effective in restoring CDI affected colonies both *in vitro* and *in vivo* ^31–33^.

In this study, ST-54 (RT 012) and ST-35 (RT 046) were the most prevalent, followed by ST-37 (RT 017) and ST-3 (RT 001), which differed from that identified by Bauer and colleagues^7^. Among the isolates obtained from the 73 hospitals in 26 countries presented by Bauer *et al*., PCR-ribotypes (RT) 014 and 001 were the most prevalent, followed by 078 and 018. Lower rates of A− B+ strains were noted in Europe^7^, while 72/307 (23.4%) of isolates were A− B+ strains in our study. The A− B+ strains appeared to be more prevalent in East Asia. For instance, several reports from Mainland China also revealed that A− B+ strains accounted for 30% of all isolates^17, 34^.

Marjolein and colleagues^6^ reported that CDT+ strains accounted for 23% (90/389) of the total isolates, and 19/90 (21.1%) of these isolates were RT 027. However, CDT+ strains in our study were rare (4.2%, 13/307), and most of the strains were ST-5 (9/13, 69.2%). Similarly, Rupnik and colleagues^35^ only found five isolates (1.6%) positive for binary toxin among the 310 isolates obtained from nine hospitals in Japan and Korea. In the present study, only a single isolate for ST-1 (RT 027) and ST-11 (RT 078) was identified, respectively, in 2012 and 2016. Due to the association of ST-1 with the CDI outbreak, the CDI incidence in the Estrie region of Quebec increased from 22.2 per 100,000 population in 1991 to 92.2 per 100,000 population in 2003^36^. This first detection of ST-1 in our hospital alerts us that this type is frequently associated with CDI outbreaks and dismal prognosis in the Western world^2^.

Furthermore, as many as 21.1% of cases were diagnosed as severe CDI, and this was in line with the reported percentages in literatures^8, 37^. Several studies had identified pulmonary infection as independent risk factors for severe CDI, and other risk factors were mentioned occasionally^27^. CDI patients that were accompanied by concomitant bloodstream and pulmonary infection may have weakened immunity, aggravating the CDI disease. High CRP likely reflects the severity of colonic inflammation, which represents a candidate marker for prognosis. Strain types have been suggested as an additional factor for CDI severity. In the present study, the disease in patients with ST-81 (5/13, 38.5%) and ST-5 (3/9, 33.3%) infection was likely more severe, compared with other STs.

Most risk factors identified in the present study for 30-day mortality were consistent with previous studies^37^. For instance, a pervious study suggested that CDI-related mortality occurred mainly within 30 days after onset, and was low after 90 days, as demonstrated by long-term follow-ups^6^. The 30-day CDI-related mortality in this cohort was 10.4%, which was comparable to the reported mortalities in North America and Europe^6, 7, 38, 39^. It was noted in our study that CDT+ strains or ST-5 infected patients were predisposed to higher mortality than other STs. Thus, different extents of pathologic changes caused by different strains may have different impacts on mortality. Interestingly, A+ B+ strains were associated with more fatal outcomes than A− B+ strains, except for ST-3. This difference in mortality between A− B+ and A+ B+ strains requires further investigation. In addition, differences in the severity of the primary disease in each CDI patient in hospitals may primarily contribute to death. Unexpectedly, we found that connective tissue disease was associated with increased mortality. Patients with connective tissue disease may have immune dysfunction, which may have made it difficult to contain CDI.

Few limitations in this study warrants discussion. First, only 30-day follow-ups were carried out, since many of the patients in this cohort resided outside of Hangzhou metropolitan boundaries. Second, our results derived a single-center study, and the main findings require verification through multicenter long-term studies in the future.

In conclusion, hospital-acquired CDI frequency in our region appears to be relatively moderate, and no clear spike was observed during the 7-year period. CDI outcomes appeared to be less severe compared to other regions. The strain types of *C. difficile* infection in our center also markedly differed from the strains reported from Western countries. We identified bloodstream infection and connective tissue disease as independent risks for severe CDI and 30-day mortality.

## Electronic supplementary material

Supplementary Table 1-3
