# Supplementary material for: Hospital-acquired Clostridium difficile infection in Mainland China: A seven-year (2009–2016) retrospective study in a large university hospital
Source: Sci Rep. 2017 Aug 29;7:9645. doi: 10.1038/s41598-017-09961-0 (PMC5575102; doi:10.1038/s41598-017-09961-0)
Supplement: Supplementary file 1 — Supplementary Table 1-3 [file 41598_2017_9961_MOESM1_ESM.doc]

**Hospital-acquired *Clostridium difficile* infection in Mainland China: A seven-year (2009-2016) retrospective study in** **a large university hospital**

**Qiaomai Xu**^a^**, Yunbo Chen**^a^**, Silan Gu**^a^**, Tao lv, Beiwen Zheng, Ping Shen, Jiazheng Quan, Yunhui Fang, and Lanjuan Li***

State Key Laboratory for Diagnosis and Treatment of Infectious Diseases, Collaborative Innovation Center for Diagnosis and Treatment of Infectious Diseases, The First Affiliated Hospital, School of Medicine, Zhejiang University, Hangzhou, Zhejiang, China

^a^ Qiaomai Xu, Yunbo Chen and Silan Gu contributed equally to this work

**Running title：Hospital-acquired *Clostridium difficile* infection in Mainland China**

***Corresponding author:** Lanjuan Li, MD, State Key Laboratory for Diagnosis and Treatment of Infectious Diseases, Collaborative Innovation Center for Diagnosis and Treatment of Infectious Diseases, The First Affiliated Hospital, School of Medicine, Zhejiang University, 79 Qingchun Road, Hangzhou 310003, Zhejiang, China.

Tel/Fax: +86 571 87236459.

E-mail: ljli@zju.edu.cn

| **Supplementary Table 1. The number of samples and patients tested per year and annual incidences of hospital-acquired *Clostridium difficile* infections** | | | | | | |
| --- | --- | --- | --- | --- | --- | --- |
| Year | No. of tested samples | No. of CDI | No. of severe CDI | No. of death | Positive (%) | Incidence (per 10,000 admissions) |
| 2009^a^ | 141 | 8 | 3 | 1 | 5.6 | 2.5 |
| 2010 | 686 | 55 | 10 | 7 | 8.0 | 4.2 |
| 2011 | 695 | 36 | 7 | 2 | 5.2 | 2.8 |
| 2012 | 542 | 36 | 10 | 2 | 6.7 | 2.8 |
| 2013 | 623 | 52 | 8 | 5 | 8.3 | 4.0 |
| 2014 | 496 | 41 | 7 | 4 | 8.3 | 3.2 |
| 2015 | 534 | 36 | 12 | 7 | 6.7 | 2.9 |
| 2016^b^ | 482 | 43 | 9 | 4 | 8.9 | 4.4 |
| ^a^ form September 1 to December 31; ^b^ form January 1 to September 30 | | | | | | |

| **Supplementary Table 2. Risk factors associated with severe hospital-acquired *Clostridium difficile* infections analyzed by univariate logistic regression** | | | | | |
| --- | --- | --- | --- | --- | --- |
| **Variable** | **OR (95% CI)** | ***P* value** | **Variable** | **OR (95% CI)** | ***P* value** |
| Age ≥65 | 2..035(1.173-3.529) | 0.011 | Mechanical ventilation prior to developing CDI | 2.869 (1.445-5.694) | 0.003 |
| Gender | 0.968 (0.531-1.765) | 0.915 | ICU admission prior to developing CDI | 2.540 (1.313-4.915) | 0.006 |
| Area | 0.903 (0.524-1.557) | 0.713 | Ward of admission at onset of CDI |  | 0.047 |
| Smoke | 0.704 (0.372-1.334) | 0.282 | Medical | Reference |  |
| Alcohol intake | 1.192 (0.627-2.266) | 0.592 | Surgical | 0.904(0.311-2.632) |  |
| Proton pump inhibitor | 0.870 (0.492-1.536) | 0.630 | Intensive care | 1.000 (0.287-3.488) |  |
| Chemotherapy | 0.619 (0.287-1.336) | 0.222 | Haemato-oncological | 2.571(0.818-8.080) |  |
| Use of any antibiotic not directed at CDI | 2.234 (0.908-5.497) | 0.080 | Geriatric | 0.783 (0.233-2.632) |  |
| Fluoroquinolones | 1.241 (0.701-2.195) | 0.459 | Any infection concomitant to CDI | 2.230 (1.284-3.875) | 0.004 |
| β-lactam/β-lactamase inhibitors combinations | 0.949 (0.521-1.729) | 0.864 | Bloodstream infection concomitant to CDI | 3.734 (1.511-9.228) | 0.004 |
| Any cephalosporin | 1.858 (1.063-3.246) | 0.030 | Pulmonary infection concomitant to CDI | 2.379 (1.343-4.212) | 0.003 |
| Carbapenem | 1.614 (0.893-2.917) | 0.113 | Other infection concomitant to CDI ^b^ | 0.526 (0.151-1.828) | 0.312 |
| Aminoglycoside | 0.000 (0.000-) | 0.999 | Fever > 38·5°C | 2.313 (1.313-4.074) | 0.004 |
| Glycopeptides | 1.625 (0.778-3.392) | 0.196 | Haemoglobin <100 g/dL | 1.506 (0.867-2.615) | 0.146 |
| Macrolide | 2.479(0.406-15.154) | 0.326 | Leukocyte ≥15× 10^9^/L | 3.125 (1.397-6.991) | 0.006 |
| Co-trimoxazole | 0.311 (0.071-1.358) | 0.121 | Albumin ≤2.5 g/dL | 0.000 (0.000- ) | 0.998 |
| Other antibiotics | 1.318 (0.498-3.487) | 0.579 | Serum creatinine increase >50% | 2.218(1.059-4.642) | 0.035 |
| Antifungal agent | 1.644 (0.863-3.131) | 0.131 | C-reactive protein ≥100 mg/L | 3.352 (1.633-6.882) | 0.001 |
| Charlson comorbidity index score ≥2 | 1.250 (0.710-2.201) | 0.439 | Therapeutic management |  | 0.542 |
| Myocardial infarction | 0.000 (0.000-) | 1.000 | No therapy | Reference |  |
| Congestive heart failure | 1.852 (0.332-10.337) | 0.483 | Symptomatic treatment | 1.000 (0.240-4.167) |  |
| Chronic obstructive pulmonary disease | 1.438 (0.493-4.189) | 0.506 | Vancomycin (oral) | 1.143 (0.277-4.721) |  |
| Cerebrovascular disease | 1.014(0.362-2.846) | 0.977 | Metronidazole (intravenous or oral) | 0.939 (0.248-3.561) |  |
| Peptic ulcer disease | 0.912 (0.100-8.296) | 0.934 | Vancomycin (oral) and metronidazole (intravenous or oral) | 1.857(0.446-7.729) |  |
| Connective tissue disease | 1.045 (0.212-5.151) | 0.957 | Sequence types (STs) |  | 0.758 |
| Diabetes | 0.546 (0.233-1.278) | 0.163 | ST-1 | Reference |  |
| Chronic kidney disease | 2.226 (1.087-4.559) | 0.029 | ST-2 | 0.521 (0.146-1.854) |  |
| Dementia | 2.479 (0.406-15.154) | 0.326 | ST-3 | 0.571(0.126-2.596) |  |
| Leukemia | 0.846 (0.399-1.792) | 0.663 | ST-35 | 0.310 (0.075-1.279) |  |
| Malignant lymphoma | 0.397 (0.049-3.188) | 0.384 | ST-37 | 0.343 (0.091-1.295) |  |
| Solid tumor | 0.768 (0.475-1.731) | 0.768 | ST-54 | 0.444 (0.119-1.662) |  |
| Liver disease | 1.341 (0.735-2.447) | 0.339 | ST-5 | 0.348 (0.094-1.289) |  |
| AIDS | 0.726 (0.083-6.325) | 0.772 | ST-8 | 0.800 (0.135-4.745) |  |
| Prior hospitalization | 1.220 (0.664-2.239) | 0.522 | ST-39 | 0.686 (0.119-3.963) |  |
| Surgery prior to developing CDI | 0.633 (0.303-1.326) | 0.226 | ST-10 | 0.20 (0.019-2.118) |  |
| Abdominal surgery prior to developing CDI | 0.869(0.381-1.985) | 0.740 |  |  |  |
| OR, Odds ratio; CI, confidence interval | | | | | |

| **Supplementary Table 3. Risk factors associated with 30-day mortality analyzed by bivariate Cox proportional hazard model in hospital-acquired *Clostridium difficile* infection patients** | | | | | |
| --- | --- | --- | --- | --- | --- |
| **Variable** | **HR (95% CI)** | **P value** | **Variable** | **HR (95% CI)** | ***P* value** |
| Age ≥65 | 2.706(1.323-5.535) | 0.006 | Mechanical ventilation prior to developing CDI | 4.995(2.466-10.120) | <0.001 |
| Gender | 1.498(0.732-3.064) | 0.269 | ICU admission prior to developing CDI | 3.095(2.022-8.295) | <0.001 |
| Area | 0.568(0.278-1.161) | 0.121 | Ward of admission at onset of CDI |  | <0.001 |
| Smoke | 0.583(0.240-1.417) | 0.234 | Medical | Reference |  |
| Alcohol intake | 0.824(0.339-2.002) | 0.670 | Surgical | 0.118(0.014-1.010) |  |
| Proton pump inhibitor | 0.986(0.476-2.045) | 0.970 | Intensive care | 0.305(0.102-0.910) |  |
| Chemotherapy | 0.772(0.297-2.006) | 0.596 | Haemato-oncological | 1.557(0.555-4.368) |  |
| Use of any antibiotic not directed at CDI | 6.354(0.867-46.545) | 0.069 | Geriatric | 0.348(0.093-1.295) |  |
| Fluoroquinolones | 1.453 (0.717-2.941) | 0.300 | Any infection concomitant to CDI | 4.630(2.142-10.009) | <0.001 |
| β-lactam/β-lactamase inhibitor combinations | 0.651(0.281-1.504) | 0.315 | Bloodstream infection concomitant to CDI | 6.538(3.021-14.147) | <0.001 |
| Any cephalosporin | 1.708(0.835-3.493) | 0.143 | Pulmonary infection concomitant to CDI | 2.859(1.429-5.718) | 0.003 |
| Carbapenem | 2.384 (1.186-4.795) | 0.015 | Other infection concomitant to CDI ^b^ | 0.821(0.196-3.437) | 0.788 |
| Aminoglycoside | 0.047 (0.000-227.158) | 0.480 | Fever >38·5°C | 3.863(1.907-7.825) | <0.001 |
| Glycopeptides | 2.735 (1.265-5.911) | 0.011 | Leukocyte ≥15× 10^9^/L | 1.434(0.503-4.088) | 0.500 |
| Macrolide | 4.310 (1.029-18.045) | 0.046 | Haemoglobin <100g/dL | 1.253(0.623-2.520) | 0.526 |
| Cotrimoxazole | 0.044 (0.000-11.344) | 0.270 | Albumin ≤2.5 g/dL | 2.429(0.935-6.309) | 0.068 |
| Other antibiotics | 1.265 (0.385-4.154) | 0.698 | Serum creatinine increase >50% | 1.357(0.523-3.525) | 0.530 |
| Antifungal agent | 2.011 (0.952-4.247) | 0.067 | C-reactive protein ≥ 100mg/L | 1.341 (0.516-3.482) | 0.547 |
| Charlson comorbidity index score ≥2 | 1.153(0.564-2.359) | 0.696 | Therapeutic management |  | 0.049 |
| Myocardial infarction | 0.050(0.000- ) | 0.824 | No therapy | Reference |  |
| Congestive heart failure | 1.734(0.237-12.703) | 0.588 | Symptomatic treatment | 0.450(0.108-1.883) |  |
| Chronic obstructive pulmonary disease | 1.752 (0.534-5.751) | 0.355 | Vancomycin (oral) | 0.481(0.138-1.672) |  |
| Cerebrovascular disease | 0.391(0.053-2.865) | 0.391 | Metronidazole (intravenous or oral) | 0.087(0.009-0.833) |  |
| Peptic ulcer disease | 0.049 (0.000-) | 0.619 | Vancomycin (oral) and metronidazole (intravenous or oral) | 1.160(0.314-4.286) |  |
| Connective tissue disease | 3.777(1.150-12.402) | 0.028 | Sequence types (STs) |  | 0.731 |
| Diabetes | 0.944(0.363-2.450) | 0.905 | ST-1 | Reference |  |
| Chronic kidney disease | 1.559(0.642-3.788) | 0.327 | ST-2 | 0.435(0.080-2.378) |  |
| Dementia | 4.068(0.972-17.028) | 0.055 | ST-3 | 0.656(0.092-4.655) |  |
| Leukemia | 0.683(0.239-1.946) | 0.475 | ST-35 | 0.162(0.015-1.788) |  |
| Malignant lymphoma | 0.954 (0.130-6.897) | 0.963 | ST-37 | 0.744(0.150-3.687) |  |
| Solid tumor | 1.242(0.574-2.683) | 0.582 | ST-54 | 0.542(0.099-2.958) |  |
| Liver disease | 0.787(0.340-1.819) | 0.575 | ST-5 | 0.910(0.193-4.287) |  |
| AIDS | 0.048 (0.000-) | 0.586 | ST-8 | 1.473(0.207-10.456) |  |
| Prior hospitalization | 1.896(0.780-4.606) | 0.158 | ST-39 | 0.602(0.055-6.639) |  |
| Surgery prior to developing CDI | 0.388(0.118-1.274) | 0.119 | ST-10 | 1.355(0.191-9.619) |  |
| Abdominal surgery prior to developing CDI | 0.417(0.100-1.747) | 0.232 |  |  |  |
| HR, hazard ratio; CI, confidence interval | | | | | |
